# Sitting Erect

**Published:** 1870-04

**Authors:** 


### SITTING ERECT.

Very many persons, in taking a seat, use the edge of it only, with the result that within a very short ten minutes they seem to be doubling up, the head bending forward over the chest. The easiest method of sitting in an erect, graceful position, is to occupy the chair in such a way that the lowest part of the spine or back-bone should touch the back of the seat, then by causing the shoulders also to touch the back, the whole body is erect, and maintains itself not only without an effort, but with a feeling of support relief, and rest, and that too without any appreciable effort. Any position maintained by the aid of a brace is forced, and is secured by an injury done to some other part which not only has to support itself, but aid in supporting parts not intended, hence must, in time, itself need aid from other sources, and thus the mischief goes on.

